# Outcomes and Incidence of PF-ILD According to Different Definitions in a Real-World Setting

**DOI:** 10.3389/fphar.2021.790204

**Published:** 2021-12-17

**Authors:** Sebastiano Emanuele Torrisi, Nicolas Kahn, Julia Wälscher, Markus Polke, Joyce S. Lee, Philip L. Molyneaux, Francesca Maria Sambataro, Claus Peter Heussel, Carlo Vancheri, Michael Kreuter

**Affiliations:** ^1^ Center for Interstitial and Rare Lung Diseases, Pneumology, Thoraxklinik, University of Heidelberg and German Center for Lung Research, Heidelberg, Germany; ^2^ Regional Referral Centre for Rare Lung Diseases, A.O.U. Policlinico-San Marco, Department of Clinical and Experimental Medicine, University of Catania, Catania, Italy; ^3^ Anschutz Medical Campus, Department of Medicine, University of Colorado Denver, Aurora, CO, United States; ^4^ National Heart and Lung Institute, Imperial College London, London, United Kingdom; ^5^ Royal Brompton Hospital, London, United Kingdom; ^6^ Radiology, Thoraxklinik, University of Heidelberg and German Center for Lung Research, Heidelberg, Germany

**Keywords:** PF-ILD, fibrosing interstitial lung disease, unclassifiable idiopathic interstitial pneumonia, CTD-ILD; connective tissue disease, non specific interstitial pneumonia

## Abstract

**Background:** Almost one-third of fibrosing ILD (fILDs) have a clinical disease behavior similar to IPF, demonstrating a progressive phenotype (PF-ILD). However, there are no globally accepted criteria on the definition of a progressive phenotype in non-IPF fILD yet. Four different definitions have been used; however, no internationally accepted definition currently exists.

**Research Question:** To compare the clinical and functional characteristics of progressive fILD according to the currently available definitions.

**Study design and methods:** Cases of fILD were identified retrospectively from the database of the tertiary referral center for ILD in Heidelberg. Lung function, clinical signs of progression, and radiological changes were evaluated. Patients with fILD were considered to have progression according to each of the four available definitions: Cottin (CO), RELIEF (RE), INBUILD (IN), and UILD study. Lung function changes, expressed as mean absolute decline of FVC%, were reported every 3 months following diagnosis and analyzed in the context of each definition. Survival was also analyzed.

**Results:** A total of 566 patients with non-IPF fILD were included in the analysis. Applying CO-, RE-, IN-, and UILD-definitions, 232 (41%), 183 (32%), 274 (48%), and 174 (31%) patients were defined as PF-ILD, respectively. RE- and UILD-criteria were the most stringent, with only 32 and 31% patients defined as progressive, while IN- was the most broad, with almost 50% of patients defined as progressive. CO- definition was in-between, classifying 41% as progressive. PF ILD patients with a UILD definition had worse prognosis.

**Interpretation:** Depending on the definition used, the existing criteria identify different groups of patients with progressive fILD, and this may have important prognostic and therapeutic implications.

## Introduction

Interstitial lung diseases (ILD) encompass a heterogenous group of parenchymal lung disorders of which many have a chronic course (Travis et al., 2013). Idiopathic pulmonary fibrosis (IPF) is the most frequent form of fibrosing ILD (fILDs) and carries the worst prognosis (Raghu et al., 2018). Of the remaining non-IPF fILDs, between 18 and 32% have a clinical behavior similar to IPF, demonstrating a progressive phenotype (PF-ILD) (Wijsenbeek et al., 2019). There are some host and disease factors that may predispose patients to be at higher risk of progression, such as the presence of a usual interstitial pneumonia (UIP) pattern, extensive ILD, or traction bronchiectasis (George et al., 2020). However, there are no globally accepted criteria on the definition of a progressive phenotype in non-IPF fILD yet (Cottin et al., 2018; Cottin, 2019). Currently, patients are defined as having a progressive phenotype if the disease progresses despite “appropriate management” (George et al., 2020). As in IPF, the decline of forced vital capacity (FVC) and/or of diffusing capacity of the lung for carbon monoxide (DLCO), the worsening of high-resolution computed tomography (HRCT) features and patient reported symptoms, have all been suggested as possible criteria to identify PF-ILD patients (Spagnolo et al., 2019). To date, four different definitions have been used to identify PF-ILD patients: one suggested by Cottin (CO) et al. in a recent review and three others based on clinical trials in PF-ILD (RELIEF [RE], INBUILD [IN], and pirfenidone in unclassifiable ILD [UILD]) (Cottin et al., 2018; Guenther et al., 2019; Wells et al., 2020; Maher et al., 2020), but no internationally accepted definition currently exists.

Corticosteroids and immunosuppressants are used as first line therapies in many non-IPF fILDs, sometimes with unpredictable and disappointing outcomes, including progressive disease behavior despite medical therapy (Flaherty et al., 2019; Kolb and Vašáková, 2019; Torrisi et al., 2019; Wong et al., 2020). Based on this clinical observation, antifibrotic drugs were suggested as a possible therapeutic strategy (George et al., 2020). The INBUILD study investigated the use of nintedanib in PF-ILD and has recently demonstrated a benefit from this treatment in patients with PF-ILD in a large range of fILDs including chronic hypersensitivity pneumonitis, autoimmune ILDs, idiopathic non-specific interstitial pneumonia (NSIP), unclassifiable ILD (uILD), and other fILDs (Wells et al., 2020). Similarly, pirfenidone was studied in the German Center for Lung Research RELIEF trial in patients with a comparable patient cohort with progressive fibrosis as in the INBUILD trial and in the uILD study (Guenther et al., 2019; Maher et al., 2020).

The clinical impact of these methods to define a progressive phenotype has not been studied outside of the clinical trial environment. We therefore aimed to compare the clinical and functional characteristics of progressive fILD in a prospective ILD registry according to the four proposed definitions of progressive disease.

## Methods

The prospective CO-WORKER in house registry of patients with ILDs in our tertiary referral center for ILD in Heidelberg was reviewed retrospectively for patients with a diagnosis of fILD other than IPF between March 2010 and November 2019 after a multidisciplinar (MDT) evaluation (pulmonologists, radiologists, pathologists, and rheumatologists). The ethics committee of the University of Heidelberg approved the retrospective data analyses (S-318/2013).

Demographic variables, medical history, functional data (FVC, DLCO, 6-min walk distance), serologic data, incidence of exacerbations (AE), and/or hospitalizations, comorbidities, and pharmacologic treatments were collected. Patients underwent routine follow-up visits including interrogation of worsening symptoms, lung function tests every 3–6 months, and radiological evaluation with HRCT every 12 months that were discussed in the context of a multidisciplinary team.

Lung function (FVC% predicted and DLCO% predicted), clinical signs of progression, and radiological changes were retrospectively evaluated. Patients with fILD were considered to have progression according to each of the four available definitions as follows:

1) Cottin (CO): any of the following criteria within a 24-month period: an absolute decline of ⩾10% in FVC; an absolute decline of ⩾15% in DLCO; or worsening symptoms or a worsening radiological appearance accompanied by a ⩾5–<10% relative decrease in FVC (Cottin et al., 2018);
2) RELIEF (RE): annualized percent predicted FVC decline ⩾5% (absolute) (within 6–24 months) (Guenther et al., 2019);
3) INBUILD (IN): any of the following criteria within a 24-month period: a relative decline in the FVC of at least 10% of the predicted value, a relative decline in FVC of 5% to less than 10% of the predicted value and worsening of respiratory symptoms or an increased extent of fibrosis on HRCT, or worsening of respiratory symptoms and an increased extent of fibrosis (Wells et al., 2020); and
4) Pirfenidone in uILD (UILD): either a more than 5% absolute decline in percent predicted FVC or significant symptomatic worsening not due to cardiac, pulmonary (except worsening of underlying unclassifiable ILD), vascular, or other causes (as determined by the investigator) within the previous 6 months (Maher et al., 2020).

Accordingly, four different groups of PF-ILD were obtained: COTTIN PF-ILD (CO-PF-ILD), RELIEF PF-ILD (RE-PF-ILD), INBUILD PF-ILD (IN-PF-ILD), and UILD PF-ILD (UILD-PF-ILD).

### Statistical Analysis

Characteristics of the study population were expressed as mean ± standard deviation, median and interquartile range, or as percentage of the relative frequency as appropriate. Baseline characteristics of each PF-ILD group were compared. A *t*-test was used to assess differences in means for continuous variables while a chi-square test was used for categorical variables. Lung function changes, expressed as mean absolute decline of FVC%, were reported every 6 months following diagnosis and analyzed in the context of each PF-ILD group. T-test was used to assess differences in mean FVC decline between each follow-up time. Univariate and multivariate Cox proportional hazard regression analysis were performed to assess Hazard Ratios for predictors of survival. Kaplan-Meier survival analysis was used to assess overall survival. Survival of the different groups was analyzed and compared with a cohort of 392 patients with a diagnosis of IPF matched for FVC% (*p* = 0.21) and DLCO% (*p* = 0.83). All the statistical analyses were performed using STATA/IC 14.2 version. A *p*-value less than 0.05 was considered significant.

## Results

### Patient Characteristics

We identified 566 patients with non-IPF fILD: 201 (35.51%) with fibrosing chronic hypersensitivity pneumonitis (cHP), 78 (13.78%) with idiopathic non-specific interstitial pneumonia (iNSIP), 111 (19.61%) with unclassifiable interstitial lung disease (uILD), 162 (28.62%) with connective tissue disease/rheumatoid arthritis associated interstitial lung diseases (CTD-ILD), and 14 (2.47%) other fILDs. The mean age was 66 ± 12 years and the majority of patients were male and former smokers. The mean FVC% predicted was 72.67 ± 21.10, while the mean DLCO % predicted was 47.07 ± 16.99. Median follow-up time was 29 months (Table 1).

**TABLE 1:** Baseline characteristics of the patients.

	fILD (*n* = 566)	PF-ILD COTTIN definition (*n* = 232) (CO)	PF-ILD RELIEF definition (*n* = 183) (RE)	PF-ILD INBUILD definition (*n* = 274) (IN)	PF-ILD uILD study definition (*n* = 174) (UILD)	CO vs RE	CO vs IN	RE vs IN	CO vs UILD	RE vs UILD	IN vs UILD
Age at diagnosis (years)	66.02 ± 11.89	66.81 ± 10.88	66.38 ± 10.43	65.81 ± 11.35	67.12 ± 10.71	0.68	0.31	0.58	0.78	0.51	0.22
Male_%	53.32	53.02	54.10	55.47	58.62	0.82	0.58	0.77	0.26	0.74	0.51
BMI	28.32 ± 5.28	28.94 ± 5.42	29.08 ± 5.28	29.07 ± 5.38	29.13 ± 5.61	0.78	0.78	0.97	0.73	0.93	0.90
Never smoker_%	41.6	41.7	36.57	38.11	37.06	0.37	0.72	0.58	0.64	0.77	0.97
Former smoker_%	53.92	53.36	60	56.6	57.65	0.37	0.72	0.58	0.64	0.77	0.97
Current Smoker_%	4.48	4.93	3.43	5.28	5.29	0.37	0.72	0.58	0.64	0.77	0.97
Biopsy_%	259 (45.76)	111 (47.84)	84 (45.9)	129 (47.08)	75 (45.4)	—	—	—	—	—	—
SLB (n, %)	24 (4.24)	10 (4.31)	11 (6.01)	13 (4.74)	10 (5.75)	—	—	—	—	—	—
Cryobiopsy (n, %)	135 (23.85)	57 (24.57)	49 (26.78)	69 (25.18)	46 (26.44)	—	—	—	—	—	—
TBB (n, %)	100 (17.67)	44 (18.97)	24 (13.11)	47 (17.15)	23 (13.22)	—	—	—	—	—	—
Bronchoalveolar lavage											
Macrophages %	73 (56,81)	72 (55,81)	74 (56,81)	73 (56,81)	75 (55,82)	—	—	—	—	—	—
Lymphocytes %	12 (4,24)	12 (4,24.5)	12 (5,24)	12 (4,24)	12 (4,23)	—	—	—	—	—	—
Neutrophils %	5 (2,10)	4 (2,10)	5 (2,10)	5 (2,10)	4 (2,10)	—	—	—	—	—	—
Eosinophils %	3 (1,7)	3 (1,7)	3 (1,7)	3 (1,7)	3 (1,7)	—	—	—	—	—	—
Mast-cells %	0 (0,1)	0 (0,1)	0 (0,1)	0 (0,1)	0 (0,1)	—	—	—	—	—	—
FVC%	72.67 ± 21.10	76.93 ± 21.60	80.03 ± 22.33	76.41 ± 21.16	75.67 ± 22.81	0.15	0.78	0.08	0.57	0.06	0.72
DLCO%	47.07 ± 16.99	48.06 ± 16.89	50.48 ± 16.72	48.86 ± 16.22	47.45 ± 16.07	0.15	0.59	0.31	0.71	0.08	0.38
6MWT distance	364 ± 106	374 ± 101	379 ± 97	375 ± 101	368 ± 102	0.63	0.89	0.71	0.61	0.34	0.51
Median follow up time (months)	29 (10,45)	34 (21,50)	32 (22,44.5)	33 (22,50)	29 (17,43)	0.39	0.94	0.42	0.01	0.08	0.01
Acute exacerbations_%	18	26.9	24.59	25.55	25.14	—	—	—	—	—	—
Background treatment											
corticosteroids (n, %)	338 (59.71)	162 (69.82)	126 (68.85)	191 (69.70)	115 (66.09)	—	—	—	—	—	—
azathioprine (n, %)	180 (31.80)	97 (41.81)	79 (43.16)	116 (42.33)	63 (36.20)	—	—	—	—	—	—
methotrexate (n, %)	88 (15.54)	49 (21.12)	37 (20.21)	49 (17.88)	27 (15.51)	—	—	—	—	—	—
cyclophosphamide (n, %)	39 (6.89)	18 (7.75)	15 (8.19)	20 (7.29)	12 (6.89)	—	—	—	—	—	—
mycophenolate mofetil (n, %)	6 (1.06)	3 (1.29)	6 (3.27)	4 (1.45)	2 (1.14)	—	—	—	—	—	—
sulfasalazine (n, %)	8 (1.41)	4 (1.72)	1 (0.54)	4 (1.45)	2 (1.14)	—	—	—	—	—	—
acetylcysteine (n, %)	59 (10.42)	28 (12.06)	15 (8.19)	32 (11.67)	24 (13.79)	—	—	—	—	—	—
pirfenidone (n, %)	23 (4.06)	19 (8.18)	13 (7.10)	18 (6.56)	10 (5.74)	—	—	—	—	—	—
nintedanib (n, %)	14 (2.47)	9 (3.87)	9 (4.91)	11 (4.01)	4 (2.29)	—	—	—	—	—	—
Underlying diagnosis											
cHP (n, %)	201 (35.51)	38.36	36.61	36.13	37.36	0.59	0.86	0.92	0.22	0.59	0.34
iNSIP (n, %)	78 (13.78)	11.64	15.30	14.6	16.67	0.59	0.86	0.92	0.22	0.59	0.34
uILD (n, %)	111 (19.61)	23.28	23.50	22.63	27.59	0.59	0.86	0.92	0.22	0.59	0.34
CTD-ILD (n, %)	162 (28.62)	25.43	21.86	24.82	17.24	0.59	0.86	0.92	0.22	0.59	0.34
Other fILD (n, %)	14 (2.47)	1.29	2.73	1.82	1.15	0.59	0.86	0.92	0.22	0.59	0.34

**FIGURE 1:**
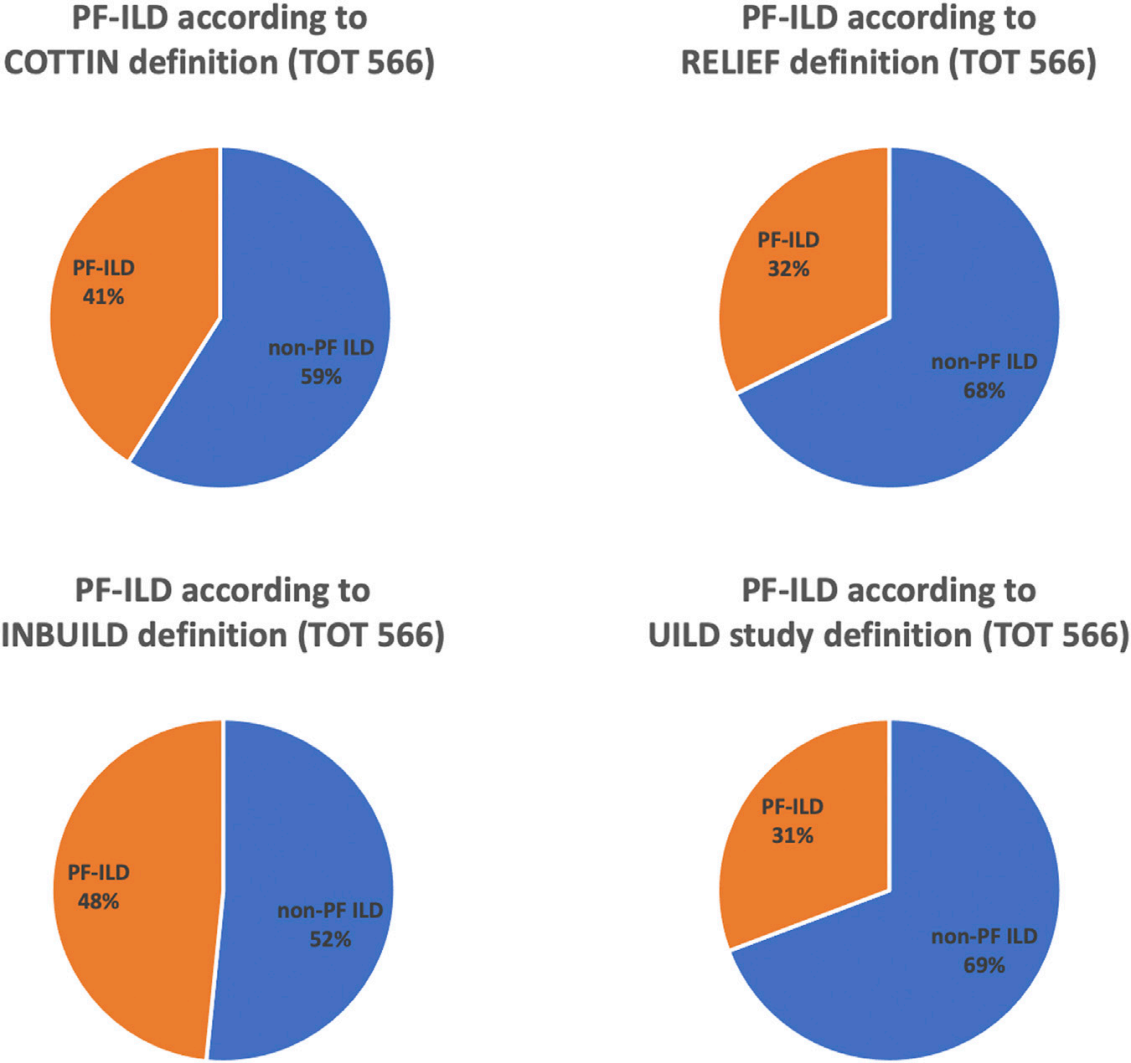
Prevalence of PF-ILD according to COTTIN, RELIEF, INBUILD, and UILD study definitions.

**FIGURE 2:**
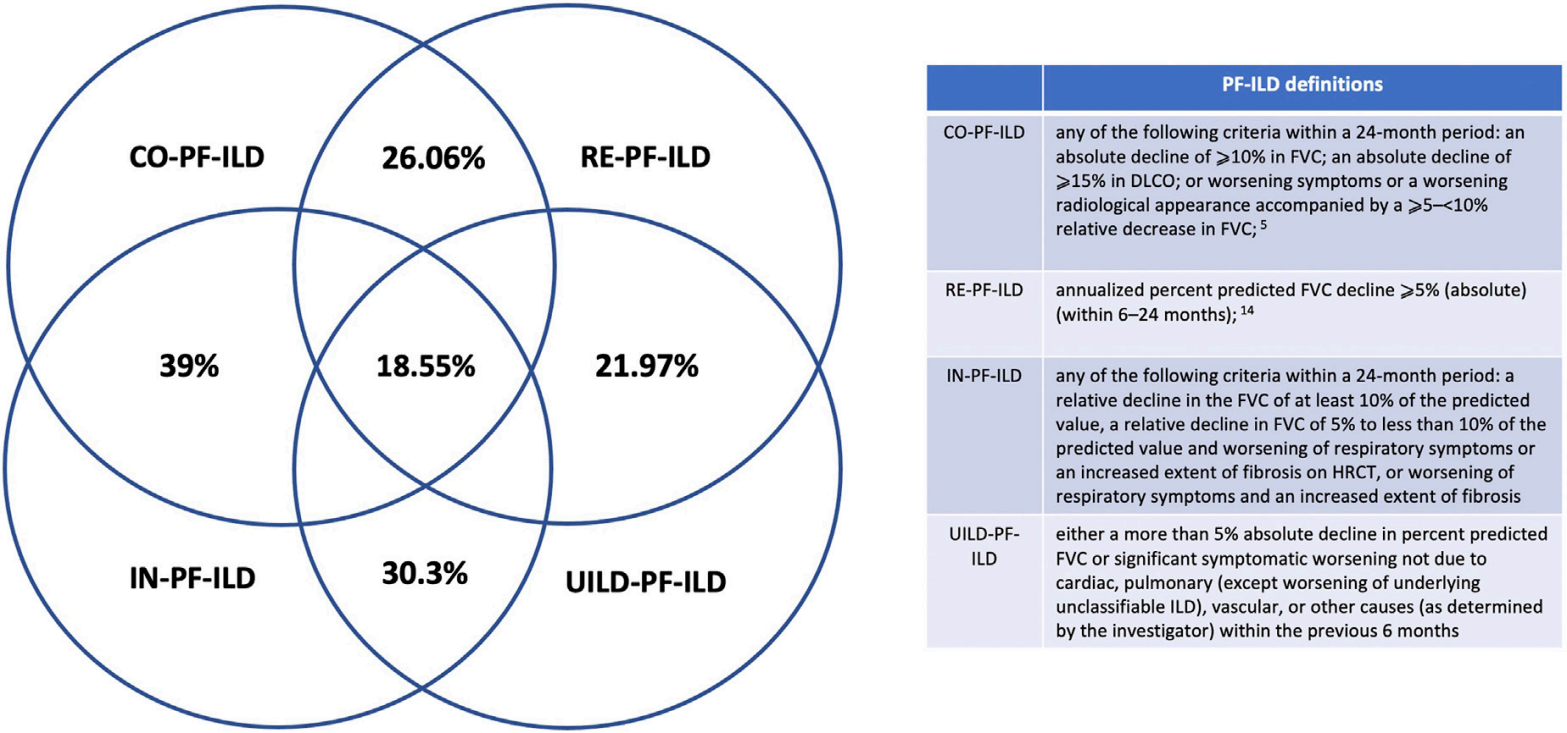
PF-ILD definitions.

Applying CO-, RE-, IN-, and UILD-definitions, 232 (41%), 183 (32%), 274 (48%), and 174 (31%) patients were defined as PF-ILD respectively (Figure 1). The prevalence of subtypes of fILD was comparable between definitions and the baseline characteristics of each PF-ILD group are listed in Table 1. Acute exacerbations (AE) were reported in the total cohort in 18% of fILD patients and in 26.9, 24.5, 22.5, and 25.1% of PF-ILD according to each definition (Table 1). Only 27% of patients met both CO- and RE-criteria, 32% met IN- and RE-criteria, and 22% met both UILD- and RE-criteria. A small group of patients (18.5%) met all four definitions (Figure 2).

### Outcomes

**FIGURE 3:**
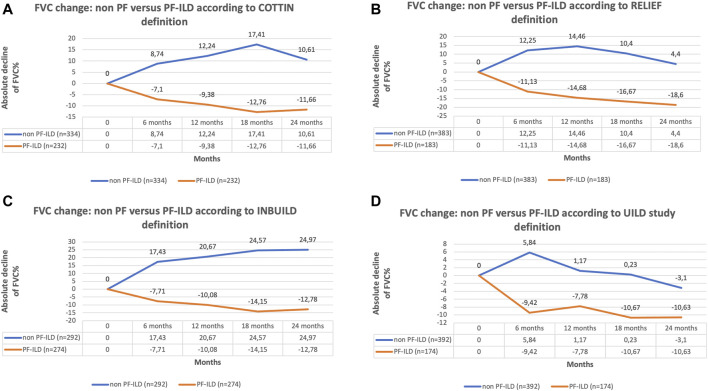
**(A)** FVC change of non PF versus PF-ILD according to COTTIN definition; **(B)** FVC change of non PF versus PF-ILD according to RELIEF definition; **(C)** FVC change of non PF versus PF-ILD according to INBUILD definition; **(D)** FVC change of non PF versus PF-ILD according to UILD study definition.

**FIGURE 4:**
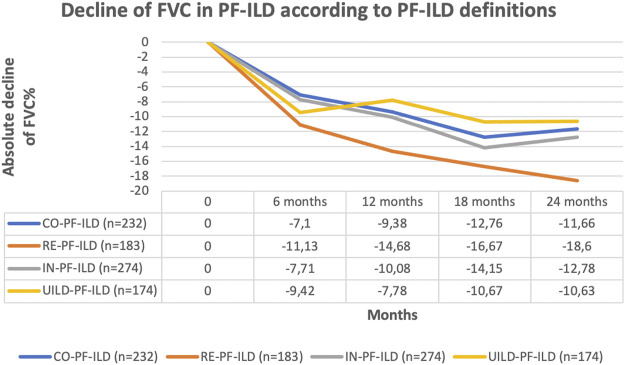
Comparison of FVC decline according to all four PF-ILD definition.

The classification of patients with fILD as progressive was associated with a significant decline in FVC compared to non-progressive fILD patients, at all time points and regardless of the definition applied (*p* < 0.0001) (Figure 3). Comparing the absolute decline of FVC according to all four PF-ILD definitions we observed a significant difference in FVC decline (*p* = 0.007 and *p* = 0.006) after 12 and 24 months comparing patients that were diagnosed as PF-ILD according to the RE-PF-ILD definition with other definitions. Disease behavior among the other groups was comparable (Figure 4).

**FIGURE 5:**
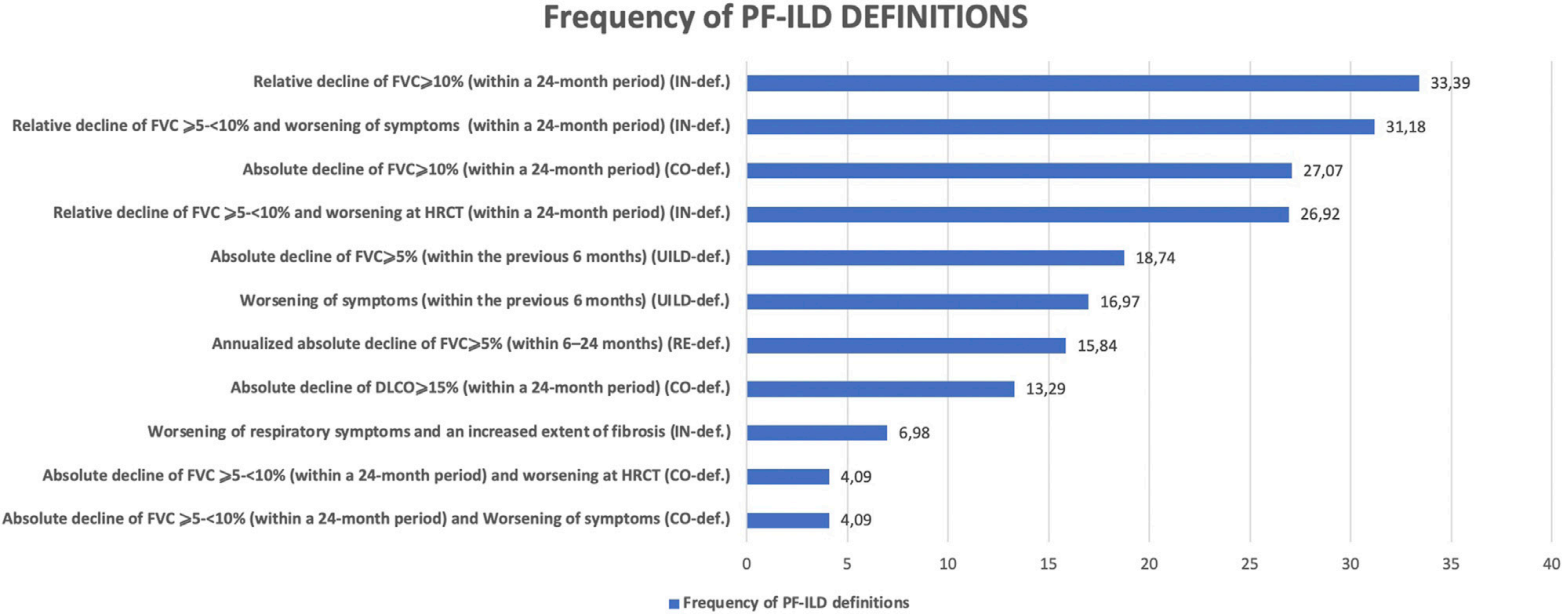
Frequency of PF-ILD definitions.

The frequency of the deconstructed criteria within the PF-ILD definitions is reported in Figure 5.

**FIGURE 6:**
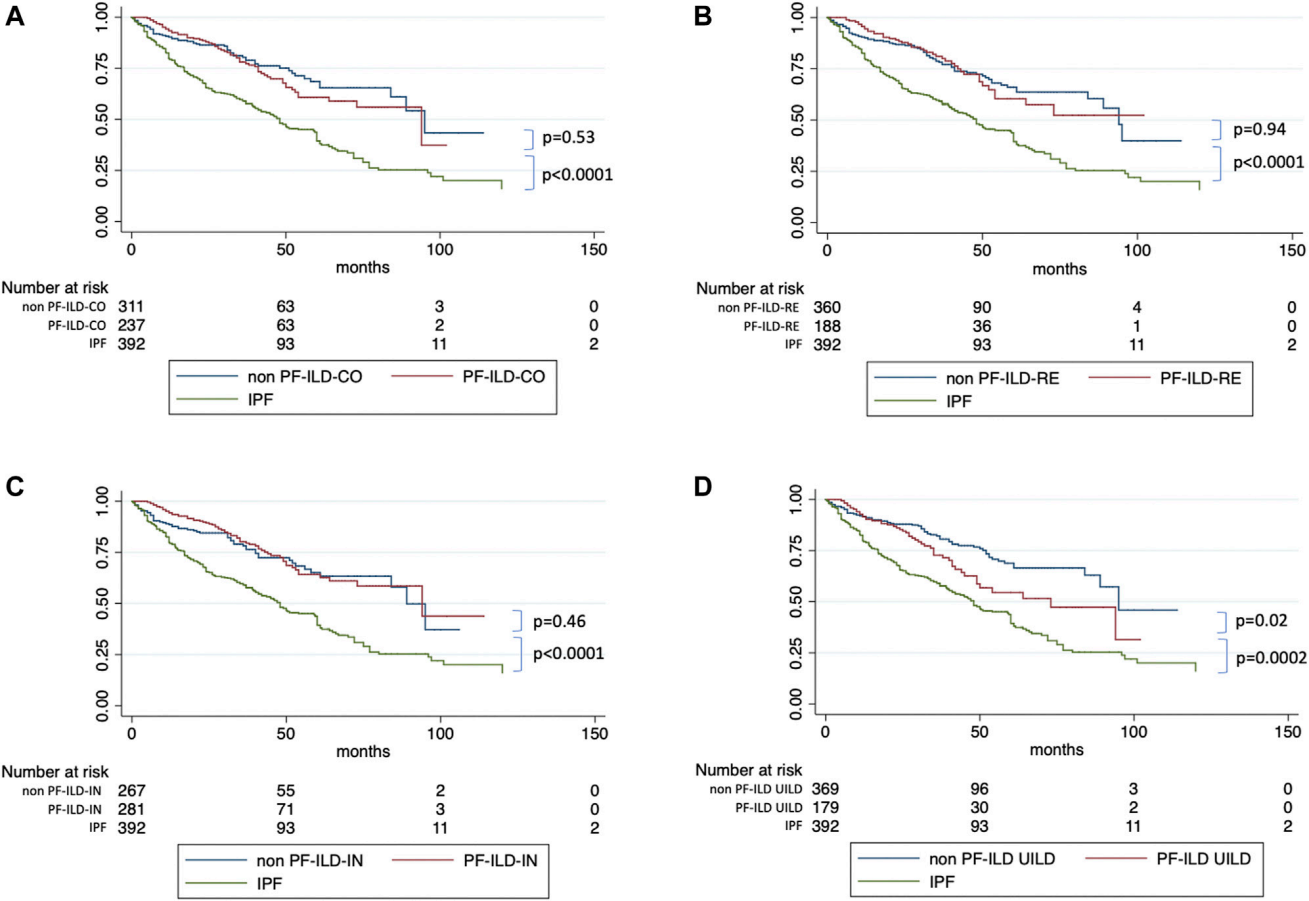
Survival of PF-ILD versus non PF-ILD compared to IPF, according to each definitions.

**FIGURE 7:**
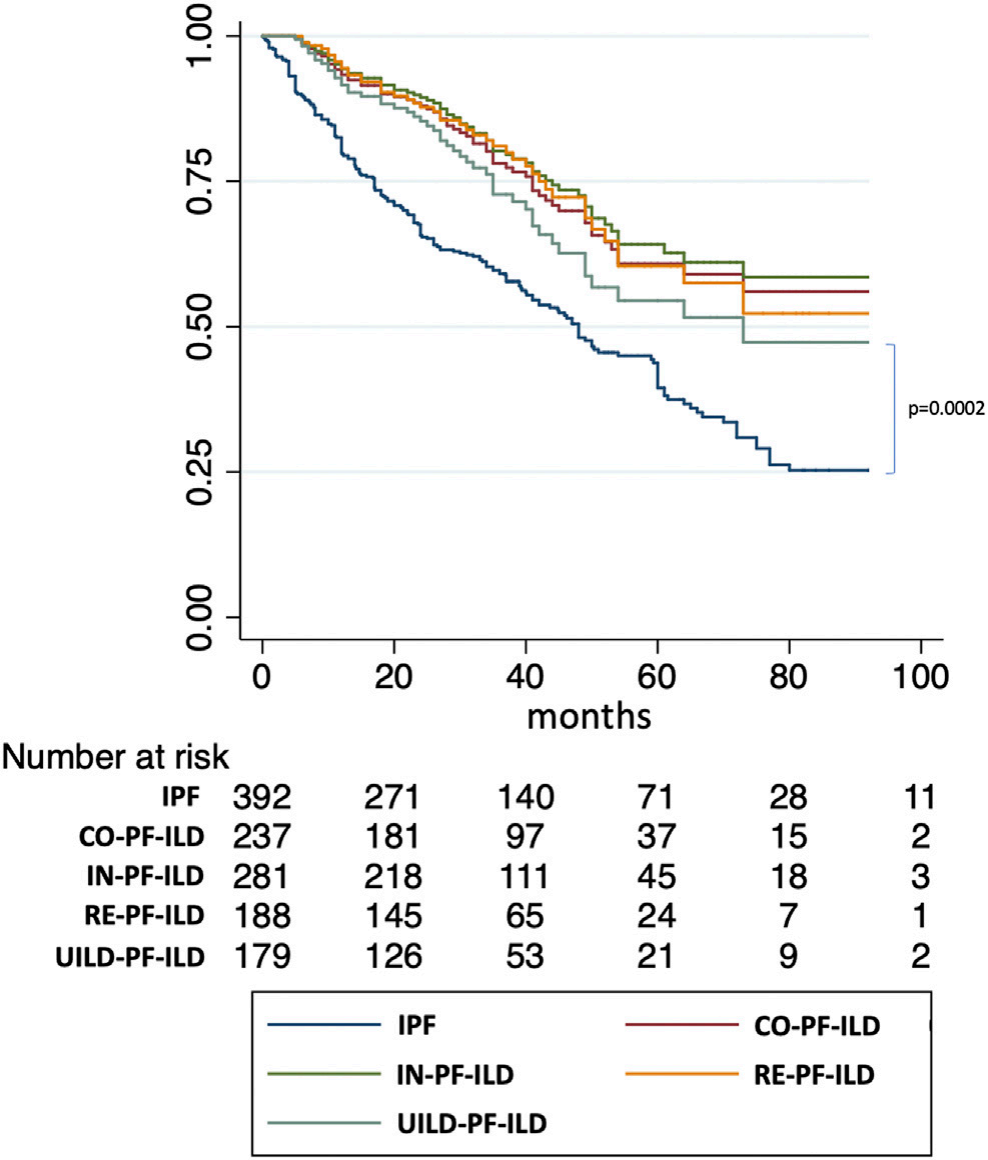
Comparison of survival of PF-ILD definitions with IPF.

We then used univariate and multivariate analyses to assess the impact of individual components of the various PF-ILD definitions on mortality (Table 2). On univariate analysis, the presence of an “absolute decline of FVC ⩾5-<10% (within a 24-month period) and worsening of symptoms,” of a “worsening of respiratory symptoms and an increased extent of fibrosis,” and of a “worsening of symptoms within the previous 6 months” demonstrated a Hazard Ratio (HR) of 2.34 (95% confidence interval, 1.28–4.24; *p* = 0.0005), 2.78 (95% confidence interval, 1.73–4.45; *p* < 0.0001), and 2.13 (95% confidence interval, 1.43–1.17; *p* < 0.0001). However, on multivariate analysis, only the presence of “worsening of symptoms within the previous 6 months” was significant with a HR of 4.45 (95% confidence interval, 1.14–17.34; *p* = 0.03). Kaplan Meier curves (Figure 6) showed a significant difference in mortality comparing progressive versus stable ILD patients only in patients selected by the UILD-PF-ILD definition (*p* = 0.02). This was not observed using other PF-ILD definitions (*p* = 0.53, *p* = 0.94, *p* = 0.46). Patients with progressive fibrosis, regardless of definition, demonstrated a better prognosis compared to patients with IPF (*p* = 0.0002) (Figure 7).

**TABLE 2:** Univariate Cox regression analysis and Multivariate Cox regression analysis adjusted for age.

Univariate cox regression analysis					
	HR	St. Err	*p*	95% conf. Interval	
Relative decline of FVC⩾10% (within a 24-month period) (IN-def.)	0.86	0.16	0.43	0.59	1.24
Relative decline of FVC ⩾5-<10% and worsening of symptoms (within a 24-month period) (IN-def.)	1.03	0.19	0.84	0.72	1.49
Absolute decline of FVC⩾10% (within a 24-month period) (CO-def.)	1.01	0.19	0.93	0.69	1.48
Relative decline of FVC ⩾5-<10% and worsening at HRCT (within a 24-month period) (IN-def.)	1.03	0.19	0.85	0.71	1.50
Absolute decline of FVC⩾5% (within the previous 6 months) (UILD-def.)	1.13	0.25	0.56	0.73	1.75
Worsening of symptoms (within the previous 6 months) (UILD-def.)	2.13	0.43	<0.0001	1.43	1.17
Annualized absolute decline of FVC⩾5% (within 6–24 months) (RE-def.)	1.04	0.23	0.84	0.59	1.60
Absolute decline of DLCO⩾15% (within a 24-month period) (CO-def.)	1.01	0.24	0.08	0.64	1.61
Worsening of respiratory symptoms and an increased extent of fibrosis (IN-def.)	2.78	0.66	<0.0001	1.73	4.45
Absolute decline of FVC ⩾5-<10% (within a 24-month period) and worsening at HRCT (CO-def.)	1.51	0.50	0.21	0.79	2.82
Absolute decline of FVC ⩾5-<10% (within a 24-month period) and Worsening of symptoms (CO-def.)	2.34	0.71	0.005	1.28	4.24
**Multivariate Cox regression analysis adjusted for age**					
	**HR**	**St. Err**	***p***	**95% conf. Interval**	
Relative decline of FVC⩾10% (within a 24-month period) (IN-def.)	0.38	0.21	0.08	0.12	1.15
Relative decline of FVC ⩾5-<10% and worsening of symptoms (within a 24-month period) (IN-def.)	0.23	0.19	0.07	0.47	1.16
Absolute decline of FVC⩾10% (within a 24-month period) (CO-def.)	2.11	1.21	0.19	0.68	6.51
Relative decline of FVC ⩾5-<10% and worsening at HRCT (within a 24-month period) (IN-def.)	1.67	1.09	0.42	0.46	6.01
Absolute decline of FVC⩾5% (within the previous 6 months) (UILD-def.)	1.41	0.53	0.35	0.67	2.95
Worsening of symptoms (within the previous 6 months) (UILD-def.)	4.45	3.09	0.03	1.14	17.34
Annualized absolute decline of FVC⩾5% (within 6–24 months) (RE-def.)	0.88	0.33	0.74	0.42	1.84
Absolute decline of DLCO⩾15% (within a 24-month period) (CO-def.)	1.27	0.47	0.52	0.60	2.65
Worsening of respiratory symptoms and an increased extent of fibrosis (IN-def.)	1.17	0.67	0.77	0.38	3.64
Absolute decline of FVC ⩾5-<10% (within a 24-month period) and worsening at HRCT (CO-def.)	1.14	1.11	0.88	0.17	7.69
Absolute decline of FVC ⩾5-<10% (within a 24-month period) and Worsening of symptoms (CO-def.)	1.07	0.89	0.93	0.21	5.49

No difference in survival was reported between patients that experienced AE comparing patients classified as PF-ILD or not regardless of the definition (Supplementary Figure S1).

## Discussion

The recognition of a growing number of fILDs other than IPF that demonstrate a progressive phenotype has highlighted the need to establish specific diagnostic criteria for progressive disease (Raghu et al., 2018). Based on the similarities with IPF, analogous criteria to define progression have also been applied in PF-ILD (Spagnolo et al., 2019). As is often the case in the field of ILD, different definitions were conceived (Cottin et al., 2018; Guenther et al., 2019; Wells et al., 2020; Maher et al., 2020) providing a lack of uniform criteria to define the progressive phenotype, potentially hindering future study design and more importantly therapeutic recommendations.

Our study demonstrates that the four current definitions used in different studies lead to a meaningful difference in numbers of patients diagnosed as having a progressive fILD. These differences could have an impact upon our patients and access to therapy. While the IN-PF-ILD criteria classified more patients with progressive disease, the classification of RE-PF-ILD was better able to identify a cohort of patients at risk of subsequent greater decline in FVC. The different definitions for PF-ILD also identified those with different disease behaviors, which may have an impact to accurately prognosticate our patients.

Moreover, there was a meaningful lack of overlap between the patients identified as being progressive, e.g., of the CO-PF-ILD patient cohort (41% of the total fILD), 14% were not included in the RE-PF-ILD group. Similarly, of the IN-PF-ILD (48% of the total fILD), 16% were not included in the RE-PF-ILD group such as in the UILD-PF-ILD (31% of the total fILD), and 9% were not included in the RE-PF-ILD. In line with this, only 18.55% of patients were included in all four definitions, highlighting again the heterogeneity of the groups identified by current PF-ILD definitions. Our results also highlight that the individual criteria that make up each of the definitions for PF-ILD also have heterogeneity in frequency and association with mortality.

Surprisingly, we observed that only patients identified using the UILD-PF-ILD definition had a difference in survival compared to non PF-ILD. This was not observed using the other definitions. These findings may be due to possible differences in genetic background, comorbidity profile, treatments effects, loss of follow-up, and other factors.

We also evaluated the possibility of including AE in the definition of disease progression. However, after analysis of our data, this was not followed further because a definition of AE-ILD is not yet available and only deducted from the 2016 statement on AE-IPF (Collard et al., 2016). Moreover, AE-ILD in non IPF might be associated with a restitutio ad integrum, and finally differences in outcome may exist for different forms of AE-IPF in IPF (Kreuter et al., 2019). Thus including AE-IPF in such a definition could bias findings.

This study has a number of strengths. Patients were evaluated in an expert center through a multidisciplinary discussion and underwent routine lung function follow-up exams every 3–6 months, reflecting real-world practice. Bronchoalveolar lavage analysis supported MDT diagnosis (e.g., CHP), even if a confident diagnosis was probably not very important with respect to the present analysis of the progressive phenotype.

However, the study also has some limitations, mainly its retrospective and single center approach. This may create some selection bias and loss to follow-up, which may affect the generalizability of the results. Second, because the data derive from real-world practice, patients were not treated in a standard manner. This made the data regarding specific treatments difficult to assess. Further, pharmacologic treatment, with either corticosteroids and immunosuppressors or a combination of these, may or may not have been given to an individual patient for a variety of reasons and differences to other cohorts regarding the use of immunosuppressive therapy may have altered outcomes.

## Conclusion

This study reveals that identifying a fILD patient as “progressive” differs depending on the definition used, which has important prognostic and therapeutic implications. Prospective and multicenter studies are needed to confirm these results and internationally accepted criteria for progression in fILD have to be defined in light of this data.

## Data Availability

The original contributions presented in the study are included in the article/Supplementary Material, and further inquiries can be directed to the corresponding author.

## Abbreviations

AE, acute exacerbations; cHP, chronic hypersensitivity pneumonitis; CTD-ILD, connective tissue diseases associated interstitial lung diseases; DLCO, diffusing capacity of the lung for carbon monoxide; fILDs, fibrosing interstitial lung diseases; FVC, forced vital capacity; HRCT; high-resolution computed tomography; ILD, interstitial lung diseases; IPF, idiopathic pulmonary fibrosis; NSIP; non-specific interstitial pneumonia; PF-ILD, progressive fibrosing interstitial lung diseases; uILD, unclassifiable interstitial lung disease; UIP, usual interstitial pneumonia.
