# Supplementary figures and images for: Outcomes and Incidence of PF-ILD According to Different Definitions in a Real-World Setting
Source: Front Pharmacol. 2021 Dec 17;12:790204. doi: 10.3389/fphar.2021.790204 (PMC8718675; doi:10.3389/fphar.2021.790204)

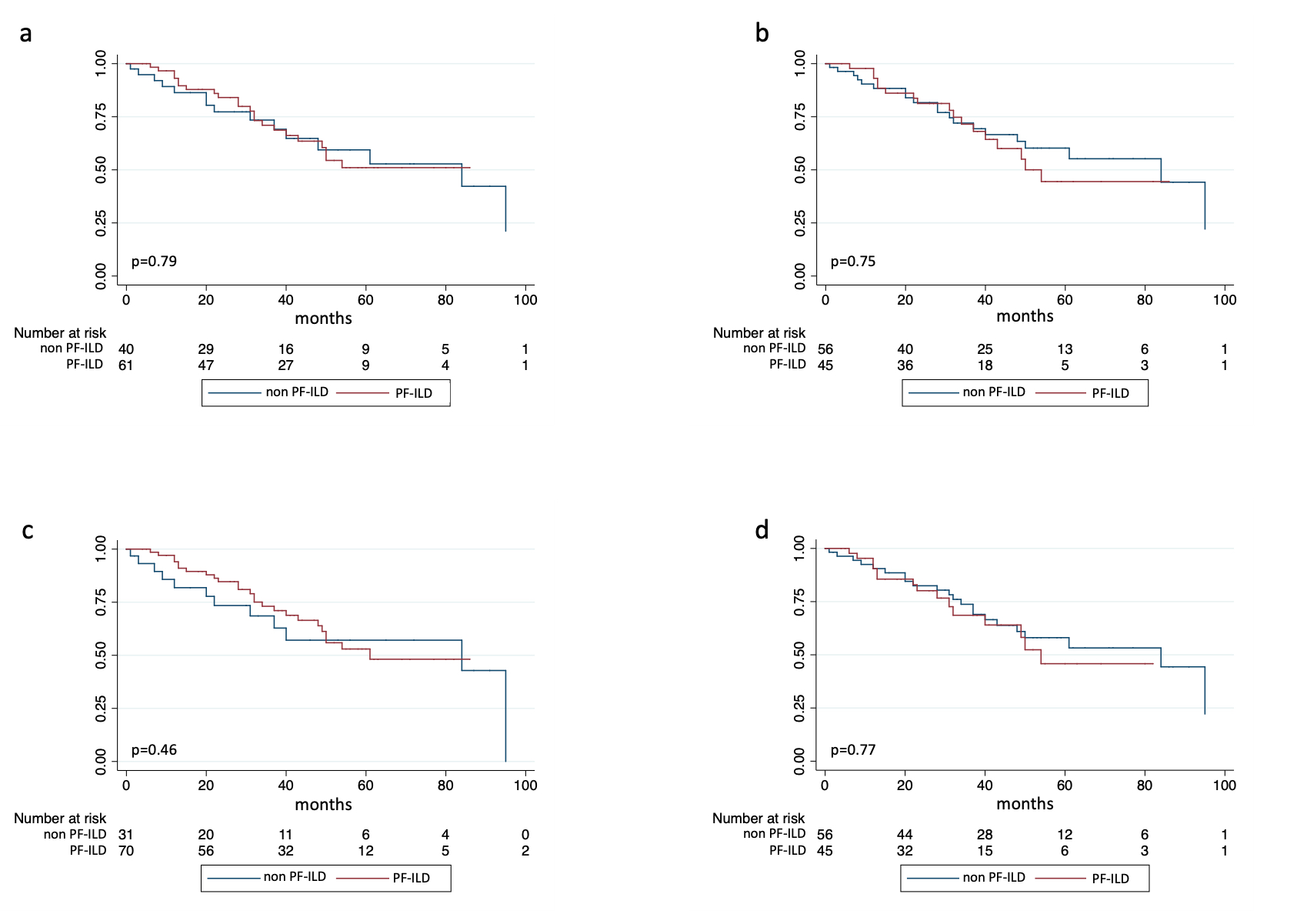

Supplement: Supplementary file 1 [file Image1.JPEG]
